# Seasonal coupling between stress physiology and gastrointestinal nematode communities in wild and semi-captive populations of an endangered ungulate

**DOI:** 10.1093/conphys/coag081

**Published:** 2026-10-22

**Authors:** Ming Lu, Yalun Wang, Hanqing Liu, Xiaofan Ma, Yongshen Wang, Yonggang Nie

**Affiliations:** Key Laboratory of Animal Biodiversity Conservation and Integrated Pest Management, Institute of Zoology, Chinese Academy of Sciences, 1 Beichen West Road, Chaoyang District, Beijing 100101, China; University of Chinese Academy of Sciences, No. 19A Yuquan Road, Shijingshan District, Beijing 100049, China; Key Laboratory of Animal Biodiversity Conservation and Integrated Pest Management, Institute of Zoology, Chinese Academy of Sciences, 1 Beichen West Road, Chaoyang District, Beijing 100101, China; Jiangxi Provincial Key Laboratory of Conservation Biology, Jiangxi Agricultural University, No. 1101 Zhimin Avenue, National Economic and Technological Development Zone, Nanchang 330045, China; Key Laboratory of Animal Biodiversity Conservation and Integrated Pest Management, Institute of Zoology, Chinese Academy of Sciences, 1 Beichen West Road, Chaoyang District, Beijing 100101, China; University of Chinese Academy of Sciences, No. 19A Yuquan Road, Shijingshan District, Beijing 100049, China; Key Laboratory of Animal Biodiversity Conservation and Integrated Pest Management, Institute of Zoology, Chinese Academy of Sciences, 1 Beichen West Road, Chaoyang District, Beijing 100101, China; University of Chinese Academy of Sciences, No. 19A Yuquan Road, Shijingshan District, Beijing 100049, China; Key Laboratory of Animal Biodiversity Conservation and Integrated Pest Management, Institute of Zoology, Chinese Academy of Sciences, 1 Beichen West Road, Chaoyang District, Beijing 100101, China; School of Life Sciences and Medical Engineering, Anhui University, No. 111 Jiulong Road, Hefei Economic and Technological Development Zone, Hefei 230601, China; Key Laboratory of Animal Biodiversity Conservation and Integrated Pest Management, Institute of Zoology, Chinese Academy of Sciences, 1 Beichen West Road, Chaoyang District, Beijing 100101, China; University of Chinese Academy of Sciences, No. 19A Yuquan Road, Shijingshan District, Beijing 100049, China

**Keywords:** Faecal cortisol, gastrointestinal nematodes, physiological stress, Przewalski’s gazelle (*Procapra przewalskii*), seasonal variation, stress–parasite interactions

## Abstract

Integrating endocrine stress biomarkers with parasite community dynamics can help clarify how environmental and management-related pressures relate to host–parasite interactions in endangered wildlife. However, relationships between physiological stress and parasite dynamics remain poorly understood, particularly in high-altitude ecosystems. Here, we investigated the endangered Przewalski’s gazelle (*Procapra przewalskii*), endemic to the Qinghai–Tibet Plateau, by combining faecal cortisol quantification with amplicon sequencing of the internal transcribed spacer 2 region of ribosomal DNA from gastrointestinal nematodes. Seasonal patterns in physiological stress and nematode community composition were compared between wild and semi-captive populations. Both faecal cortisol concentrations and nematode community composition showed pronounced seasonal variation. Trichostrongylidae reached its highest relative read abundance in summer, whereas Molineidae reached its highest relative read abundance in spring. During spring, semi-captive gazelles exhibited higher faecal cortisol concentrations and greater relative representation of Trichostrongylidae than wild gazelles. Across samples, Trichostrongylidae and Molineidae showed nearly perfectly complementary relative read abundances. Generalized linear models with a Gamma error distribution showed significant associations between faecal cortisol concentrations and the relative read abundance of each dominant nematode family after accounting for season and population type, whereas relative read abundance × population type interactions were not statistically supported. These findings reveal pronounced seasonal associations between endocrine stress responses and gastrointestinal nematode dynamics in Przewalski’s gazelle, while providing no evidence that the parasite–cortisol association differed between wild and semi-captive populations. Our study advances the integration of stress endocrinology and disease ecology in conservation physiology and supports the value of combining non-invasive endocrine and parasitological biomarkers in wildlife health assessment.

## Abbreviations

AIC, Akaike information criterion
ASV, amplicon sequence variant
GLM, generalized linear model
HPA, hypothalamic–pituitary–adrenal axis
IQR, interquartile range
ITS2, internal transcribed spacer 2
LOESS, locally estimated scatterplot smoothing
LR, likelihood ratio
NCBI, National Center for Biotechnology Information
NDVI, normalized difference vegetation index
nt, nucleotide collection
rDNA, ribosomal DNA
RIA, radioimmunoassay

## Introduction

Animals in natural ecosystems are continuously exposed to multiple external and internal stressors that challenge physiological homeostasis. External stressors include habitat fragmentation, climate change and anthropogenic disturbances (Gaynor  *et al.*, 2024; Phillips  *et al.*, 2024), whereas internal pressures may result from resource competition, social hierarchies and intra-group interactions (de  La  Peña  *et al.*, 2021). These stressors activate the hypothalamic–pituitary–adrenal (HPA) axis, stimulating the secretion of glucocorticoids that help individuals cope with environmental challenges and regulate physiological processes (Charmandari  *et al.*, 2005; Johnstone  *et al.*, 2012). However, prolonged elevation of glucocorticoid levels can lead to chronic stress, resulting in immunosuppression, metabolic dysfunction and reproductive impairment (Boonstra, 2013). Consequently, glucocorticoid concentrations have become a key physiological indicator of stress in wildlife and are widely applied in ecological and conservation physiology research (Johnstone  *et al.*, 2012; Lee  *et al.*, 2015) to quantify organismal responses to ecological disturbance and assess population-level vulnerability.

Parasitic infection represents another pervasive physiological stressor in wild animals. Parasites can impose substantial physiological costs on hosts by disrupting nutrient absorption, eliciting inflammatory responses and altering energy allocation (Colditz, 2008), while also activating complex stress responses through immune–endocrine pathways (González  *et al.*, 2020). Chronic or repeated infections may lead to persistent immune activation followed by immunosuppression (Chen  *et al.*, 2021). In ungulates, parasitic infections often exhibit marked seasonal dynamics that are closely linked to host reproductive cycles and climatic conditions (Morgan  and  Wall, 2009; Ezenwa  *et al.*, 2012; Turner  *et al.*, 2012). Such seasonal patterns have also been reported in high-altitude regions or environments characterized by strong climatic fluctuations (Yang  *et al.*, 2022), and these variations may be tightly coupled with seasonal changes in host stress physiology.

An increasing body of evidence indicates that stress hormones and parasitic infections are linked through a bidirectional regulatory relationship. On one hand, parasitic infection can activate the HPA axis via proinflammatory cytokines, leading to elevated glucocorticoid levels (Cain  and  Cidlowski, 2017; O’Dwyer  *et al.*, 2020). On the other hand, persistently high glucocorticoid concentrations can suppress immune function and increase host susceptibility to infection (Silva  *et al.*, 2016; Schoenle  *et al.*, 2019). Chronic stress may further compromise mucosal barrier integrity (Li  *et al.*, 2023), facilitating parasite invasion into host tissues. Experimental studies have demonstrated that experimentally elevated glucocorticoid exposure can increase parasite burdens in birds and mammals (Applegate, 1970; Machado  *et al.*, 2011). Collectively, these findings suggest that stress and parasitic infection may form a self-reinforcing positive feedback loop. Although this reciprocal relationship has been documented across multiple animal taxa, the underlying mechanisms in alpine ecosystems remain poorly understood, particularly for those species living in high-altitude conditions, where harsh climatic conditions and increasing human disturbance may further amplify stress–disease interactions.

Przewalski’s gazelle (*Procapra przewalskii*) is an endangered ungulate species endemic to the Qinghai–Tibet Plateau of China, a region characterized by extreme high-altitude environments with an average elevation of 4000–5000 m above sea level. Despite this, the species is currently largely confined to the Qinghai Lake basin, occurring at relatively lower elevations of ~3173–3353 m. Historically, it was widely distributed across northern provinces including Gansu, Inner Mongolia, Ningxia and Qinghai (Jiang  *et al.*, 2000). However, extensive habitat fragmentation, poaching and intensive livestock grazing have caused severe declines in both population size and distribution range (Jiang  *et al.*, 1995, 2001). Today, remnant wild populations persist in several isolated patches surrounding Qinghai Lake. These wild populations experience substantial anthropogenic disturbances such as livestock grazing and tourism, and face strong ecological pressures including food competition and human interference (Li  and  Jiang, 2008; Li  *et al.*, 2012; You  *et al.*, 2013; Zhang  *et al.*, 2013). In addition to these wild populations, a semi-captive population has been established at the Przewalski’s Gazelle Conservation and Research Center on the southern shore of Qinghai Lake. The gazelles are maintained within a large fenced enclosure (~26.27 ha) dominated by natural vegetation, with supplementary feeding and water provided only during winter (Zhang  *et al.*, 2024). The coexistence of wild and semi-captive populations under contrasting management regimes provides a comparative framework for examining how environmental and management contexts are associated with endocrine stress responses and gastrointestinal nematode community dynamics.

**Figure 1:**
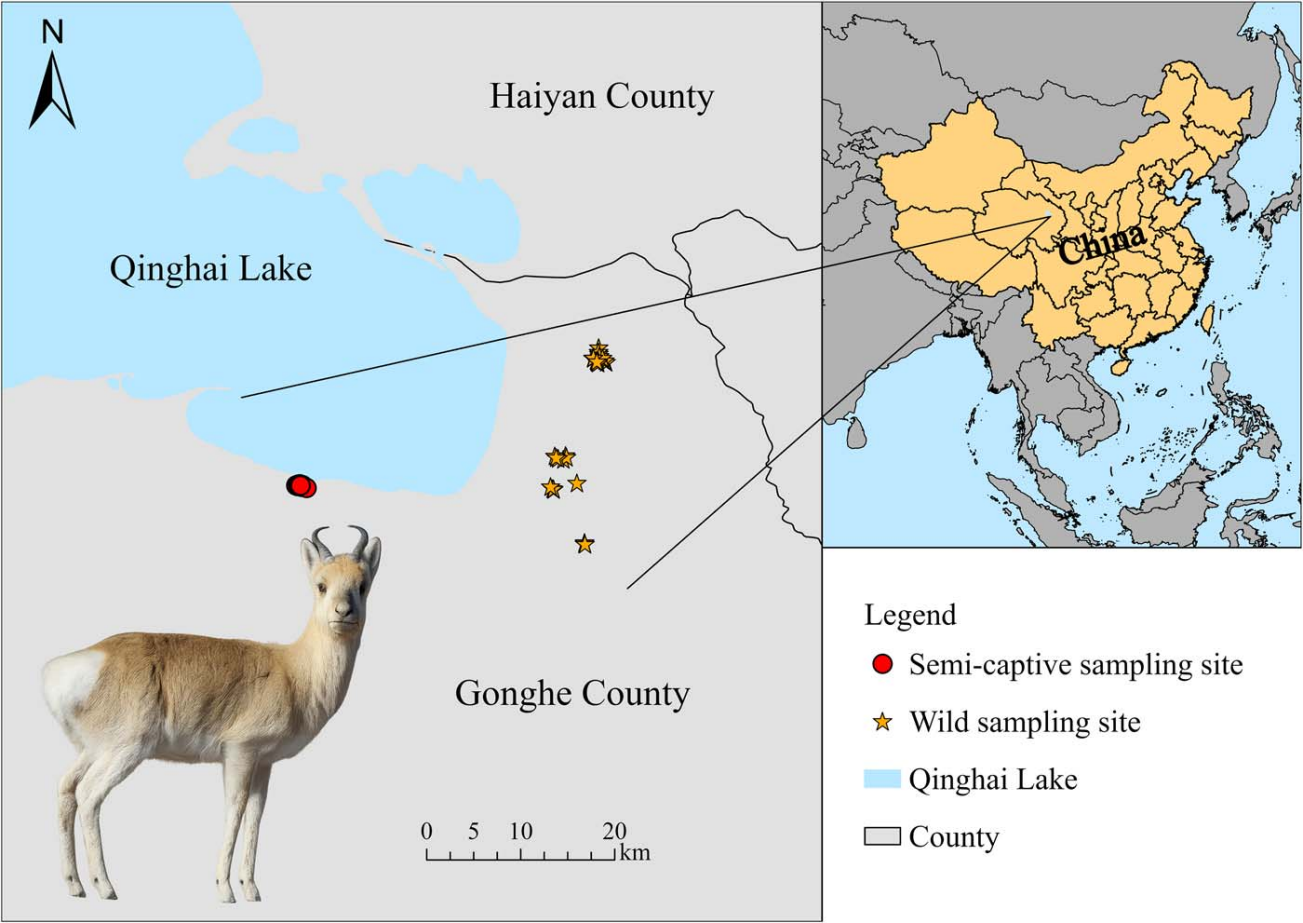
Study area and sampling sites of Przewalski’s gazelle around Qinghai Lake, China. The inset shows the location of the study area within China.

In this study, we integrated faecal cortisol assays with amplicon sequencing targeting the internal transcribed spacer 2 (ITS2) region of ribosomal DNA, a widely used molecular marker for nematode identification and characterization of community composition. We assessed seasonal and population-level variation in physiological stress and nematode community composition between wild and semi-captive populations of Przewalski’s gazelle. In addition, we examined associations between faecal cortisol concentrations and the relative read abundances of the dominant nematode families and tested whether these associations differed between population types. We hypothesized that cortisol concentrations would be associated with shifts in dominant nematode community composition and that both would vary with season and population type. By combining endocrine and parasitological measures, this study provides an integrative assessment of stress physiology and gastrointestinal nematode community dynamics in Przewalski’s gazelle and contributes to a broader understanding of stress–parasite associations in alpine ecosystems.

## Materials and methods

### Study area and populations

This study examined both wild and semi-captive populations of *P. przewalskii* inhabiting contrasting ecological settings around Qinghai Lake, China. The wild population occupies the Hudong region on the lake’s eastern shore in Qinghai Province (36°30′–36°49′ N, 100°32′–100°50′ E; elevation 3173–3353 m), an area characterized by a continental, semi-arid alpine-grassland climate. The dominant vegetation consists mainly of *Achnatherum splendens*, *Stipa penicillata* and *Stellera chamaejasme*. The field survey in 2023 estimated this local population at ~40 individuals. The semi-captive population is maintained at the Przewalski’s Gazelle Conservation and Research Center on the southern shore of Qinghai Lake. The fenced enclosure covers about 26 ha and supports vegetation similar to that of the Hudong region. During winter, the gazelles receive supplemental feed consisting primarily of oat grass (*Avena sativa*), while natural forage predominates throughout the rest of the year. The semi-captive population consisted of ~80 individuals in 2025. Both sites experience a comparable continental, semi-arid alpine climate, with a mean annual temperature of about 3°C and annual precipitation ranging from 291 to 579 mm. The geographical locations of the study areas and sampling sites are shown in Fig. 1.

### Sample collection and verification of species identity

A total of 243 fresh faecal samples of *P. przewalskii* were collected from 2023 to 2025 across four seasons: spring (March–April), summer (June–July), autumn (September–October) and winter (December–January). Among these samples, 208 were used for faecal cortisol assays and 150 for molecular analyses of gastrointestinal nematodes, with 115 samples analysed for both hormonal and parasitological parameters. Detailed sample allocation across analytical categories, seasons and population types is provided in Table 1.

**Table 1:** Number of faecal samples used for cortisol assays, ITS2 metabarcoding and combined analyses across seasons in wild and semi-captive Przewalski’s gazelle populations

**Analysis category**	**Population type**	**Spring**	**Summer**	**Autumn**	**Winter**	**Total**
	Wild	17	40	21	38	116
Cortisol assays	Semi-captive	24	23	31	14	92
	Total	41	63	52	52	208
	Wild	19	19	11	20	69
ITS2 metabarcoding	Semi-captive	22	25	20	14	81
	Total	41	44	31	34	150
	Wild	13	16	7	12	48
Combined analyses	Semi-captive	16	19	18	14	67
	Total	29	35	25	26	115

Combined analyses include paired samples for which both faecal cortisol and ITS2 metabarcoding data were available.

During sampling, Przewalski’s gazelles were observed from a distance using binoculars to identify suitable sampling locations. Fresh faecal samples were subsequently collected from recently occupied sites only after the animals had voluntarily moved away from the area without any disturbance or intervention by researchers. Fresh pellets were identified by their moist surface and shiny mucous coating. Each sample was collected using sterile disposable polyethylene gloves and tubes, temporarily stored on dry ice and transferred to a −40°C freezer on the same day for long-term preservation. Reliable individual recognition was not feasible because sampling was conducted non-invasively in free-ranging Przewalski’s gazelles, which are highly vigilant and typically observed from a distance to minimize disturbance. Furthermore, individuals lacked stable natural external characteristics that would allow consistent visual identification under field conditions. To minimize repeated sampling from the same individual, samples were collected from spatially separated locations and differentiated where possible based on faecal characteristics and basic sex identification. In addition, samples used for downstream analyses were randomly selected from the overall sample pool to reduce the probability of repeated inclusion of the same individuals.

Genomic DNA was extracted from preserved faecal samples using the SPINeasy DNA Pro Kit for Feces (MP Biomedicals, USA) following the manufacturer’s instructions. To confirm the species origin of all samples, a fragment of the mitochondrial cytochrome *b* (Cyt *b*) gene was amplified with universal mammalian primers (L14724: 5′-CGAAGCTTGATATGAAAAACCATCGTTG-3′; H15149: 5′-AAACTGCAGCCCCTCAGAATGATATTTGTCCTCA-3′). PCR conditions were: initial denaturation at 95°C for 5 min; 35 cycles of 95°C for 30 s, 55°C for 30 s, and 72°C for 30 s; followed by a final extension at 72°C for 10 min. PCR products were sequenced by SinoGenoMax (Beijing, China), and the resulting sequences were aligned against the National Center for Biotechnology Information (NCBI) nucleotide collection (nt) database using BLAST to verify that all samples originated from *P. przewalskii*.

### Faecal cortisol extraction and assay

Frozen faecal samples were thawed at 4°C before analysis. Approximately 0.3 g of wet faeces was placed into a 15-ml centrifuge tube containing 5 ml of methanol–water (9:1, v/v). The mixture was vortexed for 5 min and centrifuged at 2500 rpm for 15 min, and the supernatant was collected. The extraction procedure was repeated once, and the two supernatants were pooled and thoroughly mixed. A 1-ml aliquot of the combined extract was transferred to a new tube and stored at −20°C until analysis. Prior to assay, aliquots were oven-dried at 40°C for 12 h to constant weight.

Faecal cortisol concentrations were quantified by the Beijing North Institute of Biotechnology Co., Ltd using a high-sensitivity radioimmunoassay (RIA) kit (range = 0.313–50 ng/ml; sensitivity = 0.16 ng/ml; intra-assay coefficient of variation < 10%; inter-assay coefficient of variation < 15%). Measurements were performed with an XH6080 gamma counter (Beijing Zhongjia Scientific Instrument Co., China) designed for RIA determinations.

Faecal cortisol concentrations (ng/g dry faeces) were analysed using a generalized linear model (GLM) with a Gamma error distribution and log link function. Population type, season, sex and the population type × season interaction were included as explanatory variables. Model terms were evaluated using likelihood ratio (LR) tests. When significant effects were detected, post hoc pairwise comparisons between wild and semi-captive populations within each season were performed using estimated marginal means.

To evaluate whether broad environmental variation explained additional variation in faecal cortisol concentrations, a second Gamma GLM was fitted by adding standardized Normalized Difference Vegetation Index (NDVI), mean temperature and cumulative precipitation as covariates. The contribution of environmental covariates to model fit was assessed using LR tests. All analyses were conducted in R (R  Core  Team, 2025), with significance set at *P* < 0.05.

### Molecular detection of gastrointestinal nematodes

Gastrointestinal nematode communities were characterized by amplifying and sequencing the ITS2 region of ribosomal DNA (rDNA) (Davey  *et al.*, 2021). Three 3-g faecal subsamples were homogenized in 57 ml of water and passed through a ~1000-μm sieve. The resulting suspension was divided into three 15-ml tubes and centrifuged at 1550 × *g* for 3 min, after which the supernatants were discarded. Approximately 300 μg of the remaining pellet was transferred from one tube into a 2-ml tube, and genomic DNA was extracted using the SPINeasy DNA Pro Kit for Feces (MP Biomedicals, USA) according to the manufacturer’s instructions.

The ITS2 region of rDNA was amplified using the primers NC1 (5′-ACGTCTGGTTCAGGGTTGTT-3′) and NC2 (5′-TTAGTTTCTTTTCCTCCGCT-3′). PCR conditions consisted of an initial denaturation at 95°C for 5 min, followed by 35 cycles of 95°C for 1 min, 54°C for 1 min and 72°C for 1 min, with a final extension at 72°C for 5 min. PCR products were visualized on a 1.5% agarose gel, and successful amplicons were sequenced on the Illumina MiSeq platform (Magigene, Guangzhou, China).

### Sequence processing and taxonomic assignment

Raw sequence data from Illumina sequencing were processed using the DADA2 pipeline in R v4.4.3 (Callahan  *et al.*, 2016) for quality filtering, error correction, chimaera removal and identification of amplicon sequence variants (ASVs) by Shanghai Majorbio Bio-Pharm Technology Co., Ltd. The resulting sequences were aligned against the NCBI nt database for taxonomic annotation, and ASVs with ≥99% identity, ≥80% coverage and *E*-value ≤1 × 10^−5^ were retained for downstream analyses.

### Relative read abundance calculation and statistical analyses

Raw ASV read counts were normalized to relative read abundance values to account for variation in sequencing depth among samples. Relative read abundance was calculated as the proportion of sequencing reads assigned to each nematode taxon relative to the total nematode reads within each sample and was used to characterize gastrointestinal nematode community composition. Zero values indicated that no sequencing reads corresponding to a given taxon were detected within a sample following sequence processing and normalization. Relative read abundance values are presented as medians and interquartile ranges (IQRs). All downstream statistical analyses were performed directly on the original normalized relative read abundance values using non-parametric tests. Differences between wild and semi-captive populations within each season were evaluated using Wilcoxon rank-sum tests. Within each nematode family, *P* values from the four seasonal comparisons were adjusted using the Bonferroni correction. All statistical analyses were performed in R (R  Core  Team, 2025), with statistical significance set at *P* < 0.05.

**Table 2:** Effects of population type, season, sex and the population type × season interaction on faecal cortisol concentrations based on a Gamma GLM

**Term**	**LR *χ*** ^**2**^	***df***	***P* value**
Population type	13.04	1	<0.001
Season	94.75	3	<0.001
Sex	1.35	1	0.246
Population type × season	136.88	3	<0.001

The model used a Gamma error distribution with a log link function. Significance was assessed using LR tests. LR χ^2^, likelihood-ratio chi-square statistic; *df*, degrees of freedom.

### Associations between faecal cortisol and nematode community composition

Associations between faecal cortisol concentrations and gastrointestinal nematode community composition were assessed using samples for which both hormonal and ITS2 metabarcoding data were available (*n* = 115). Faecal cortisol concentrations were analysed using GLMs with a Gamma error distribution and log link function. Separate models were fitted for Trichostrongylidae and Molineidae, with relative read abundance as the focal predictor and season and population type (wild or semi-captive) included as covariates. To test whether the association between nematode relative read abundance and cortisol varied between population types, a corresponding interaction model including a relative read abundance × population type interaction term was fitted for each nematode family. The significance of model terms was assessed using Type II LR *χ*^2^ tests, and the added contribution of the interaction term was further evaluated by comparing the additive and interaction models using a LR test. Akaike information criterion (AIC) was used to compare model fit and parsimony. When the interaction term was not statistically supported, the more parsimonious additive model was retained for interpretation.

For exploratory visualization, scatterplots with locally estimated scatterplot smoothing (LOESS) curves and 95% confidence bands were generated using the ggplot2 package in R. These plots were used solely to visualize patterns in the data and were not used for statistical inference. All statistical analyses were performed in R (R  Core  Team, 2025), with statistical significance set at *P* < 0.05.

### Ethics statement

This study involved only the non-invasive collection of naturally voided faecal samples from wild and semi-captive Przewalski’s gazelles. No animals were captured, restrained, handled or subjected to experimental manipulation. All field sampling was conducted in accordance with relevant national and institutional guidelines for wildlife research. As the study involved no direct intervention with animals and no experimental procedures, formal animal ethics approval was not required.

## Results

### Effects of population type and season on faecal cortisol concentrations

In the Gamma GLM, both population type (LR χ^2^ = 13.04, *df* = 1, *P* < 0.001) and season (LR *χ*^2^ = 94.75, *df* = 3, *P* < 0.001) significantly affected faecal cortisol concentrations. The population type × season interaction was also highly significant (LR *χ*^2^ = 136.88, *df* = 3, *P* < 0.001; Table 2), indicating that the difference in faecal cortisol concentrations between population types varied among seasons. Sex had no significant effect (LR *χ*^2^ = 1.35, *df* = 1, *P* = 0.246).

Post hoc comparisons based on estimated marginal means showed that semi-captive gazelles had significantly higher cortisol concentrations than wild gazelles in spring (Wild/Semi-captive ratio = 0.216, *P* < 0.001; Fig. 2; Table S1). No significant population difference was detected in summer (ratio = 1.044, *P* = 0.684). In contrast, wild gazelles had significantly higher cortisol concentrations than semi-captive gazelles in autumn (ratio = 1.460, *P* = 0.001) and winter (ratio = 1.295, *P* = 0.041).

**Figure 2:**
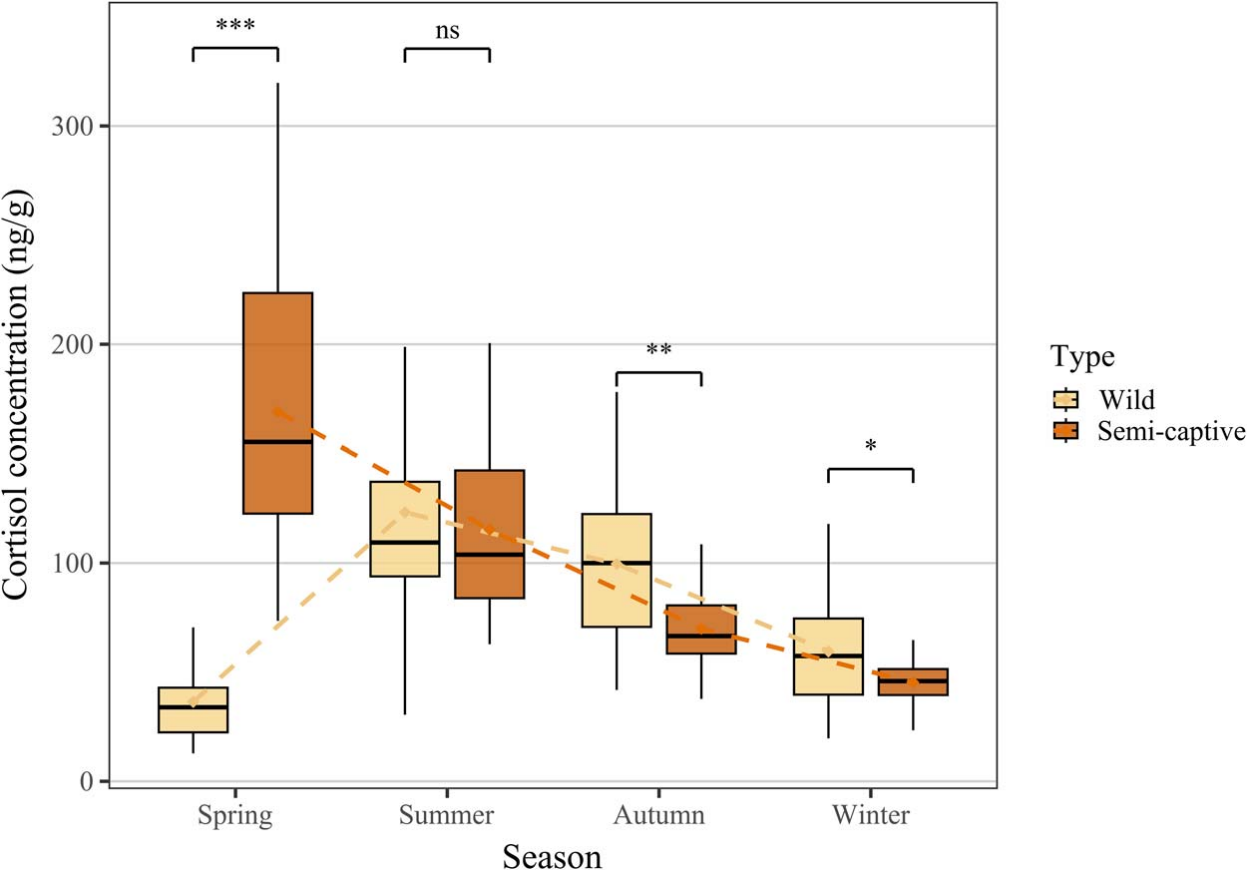
Seasonal variation in faecal cortisol concentrations across wild and semi-captive Przewalski’s gazelles. Boxplots represent the distribution of faecal cortisol concentrations, and dashed lines connect means across seasons. Asterisks indicate within-season comparisons between wild and semi-captive populations based on estimated marginal means from the Gamma GLM (^***^  *P* < 0.001; ^**^  *P* < 0.01; ^*^  *P* < 0.05; ns, not significant).

When NDVI, mean temperature and cumulative precipitation were included as environmental covariates, the population type × season interaction remained significant (LR *χ*^2^ = 117.04, *df* = 3, *P* < 0.001; Table S2). However, the independent effects of NDVI, temperature and precipitation were not significant (Table S2), and adding these environmental covariates did not significantly improve model fit relative to the base model (ΔDeviance = 1.104, *df* = 3, *P* = 0.073). These results suggest that seasonal differences in faecal cortisol concentrations were mainly associated with population type × season effects, whereas the independent effects of the measured environmental covariates were limited and should be interpreted cautiously.

**Table 3:** Nematode taxa identified from faecal samples of Przewalski’s gazelles across sampled populations and seasons

**Family**	**Genus**	**Species-level taxon**
Trichostrongylidae	*Marshallagia*	*Marshallagia dentispicularis*
Trichostrongylidae	*Ostertagia*	*Ostertagia* sp. NMG-2010
Trichostrongylidae	*Teladorsagia*	*Teladorsagia circumcincta*
Molineidae	*Nematodirella*	*Nematodirella longissimespiculata*
Molineidae	*Nematodirus*	*Nematodirus andersoni*
Molineidae	*Nematodirus*	*Nematodirus davtiani*
Molineidae	*Nematodirus*	*Nematodirus oiratianus*
Molineidae	*Nematodirus*	*Nematodirus* sp. NMG-2010
Trichostrongylidae	*Trichostrongylus*	*Trichostrongylus axei*
Trichostrongylidae	*Trichostrongylus*	*Trichostrongylus* sp. NMG-2010
Trichostrongylidae	*Trichostrongylus*	*Trichostrongylus vitrinus*
Trichuridae	*Trichuris*	*Trichuris ovis*

Taxonomic identification was based on ITS2 amplicon sequencing. Scientific genus and species names are italicized; ‘sp.’ denotes an unidentified species within the indicated genus and is not italicized.

**Figure 3:**
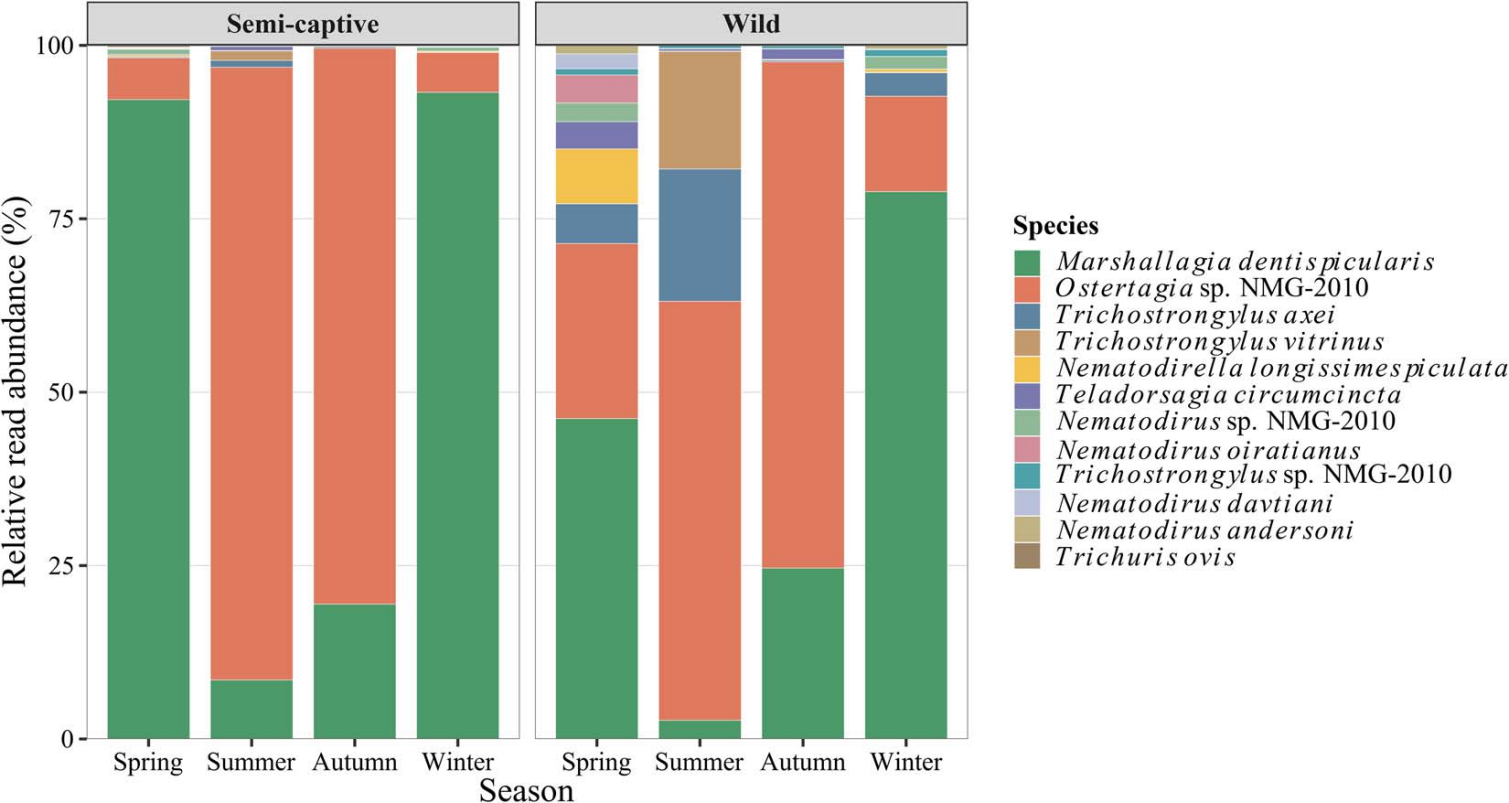
Seasonal species-level composition of gastrointestinal nematode communities in wild and semi-captive populations. Stacked bar plots illustrate the mean relative read abundance of nematode taxa within each population and season.

### Composition and variation of gastrointestinal nematode communities in Przewalski’s gazelle

A total of 12 nematode taxa, representing seven genera across three families (Trichostrongylidae, Molineidae and Trichuridae), were identified across all faecal samples (Table 3). At the family level, most nematode sequences belonged to Trichostrongylidae, which exhibited the highest mean relative read abundance (96.96%; range: 28.38%–100%). Molineidae comprised 2.99% of reads on average (range: 0%–71.58%), while Trichuridae occurred only sporadically, with a mean relative read abundance of 0.04% (range: 0%–2.99%) and was absent from several samples. Owing to its extremely low relative read abundance, Trichuridae was excluded from subsequent comparative analyses. A total of 12 ASVs were identified at the species level. Among these, *Marshallagia dentispicularis* (mean relative read abundance: 44.75%, range: 0%–99.7%) and *Ostertagia* sp. NMG-2010 (mean relative read abundance: 44.87%, range: 0%–97.92%) were the dominant taxa and together accounted for the majority of sequencing reads within the Trichostrongylidae assemblage (Fig. 3). In addition, *Trichostrongylus axei*, *Trichostrongylus vitrinus*, *Trichostrongylus* sp. NMG-2010, *Teladorsagia circumcincta* and several *Nematodirus* species, including *N. oiratianus*, *N. davtiani* and *N. andersoni*, were also detected but occurred at comparatively low relative read abundance (<10%).

The families Trichostrongylidae and Molineidae were the dominant gastrointestinal nematode groups detected, and their relative read abundances varied markedly across seasons and populations (Table 4). Trichostrongylidae was the predominant family throughout the study period and consistently accounted for the majority of nematode sequencing reads across seasons (Table 4). In both wild and semi-captive populations, the relative read abundance of Trichostrongylidae was highest in summer, with relatively high values observed in autumn and winter. Comparisons between populations showed that semi-captive gazelles exhibited significantly higher relative read abundance of Trichostrongylidae in spring (*P* < 0.001), whereas no significant differences were detected in summer (*P* = 1.000), autumn (*P* = 0.346) and winter (*P* = 1.000).

**Table 4:** Seasonal comparisons of relative read abundance of dominant gastrointestinal nematode families between wild and semi-captive populations

**Family**	**Season**	**Wild median (Q1–Q3) (%)**	**Semi-captive median (Q1–Q3) (%)**	**Adjusted *P* value**
Trichostrongylidae	Spring	90.0 (76.5–97.6)	99.0 (97.9–99.7)	<0.001
	Summer	100.0 (99.9–100.0)	100.0 (99.8–100.0)	1.000
	Autumn	99.9 (99.7–99.9)	99.9 (99.9–100.0)	0.346
	Winter	99.2 (97.8–99.8)	99.7 (98.6–99.8)	1.000
Molineidae	Spring	10.0 (2.4–23.5)	0.8 (0.2–1.3)	<0.001
	Summer	0.0 (0.0–0.1)	0.0 (0.0–0.2)	1.000
	Autumn	0.1 (0.1–0.3)	0.1 (0.0–0.1)	0.241
	Winter	0.8 (0.2–2.2)	0.3 (0.2–1.4)	1.000

Values are presented as median relative read abundance (%) with IQRs (Q1, first quartile; Q3, third quartile). Differences between wild and semi-captive populations within each season were evaluated using Wilcoxon rank-sum tests. Within each nematode family, *P* values from the four seasonal comparisons were adjusted using the Bonferroni correction. Adjusted *P* values are shown.

**Table 5:** Additive Gamma GLMs of associations between faecal cortisol concentrations and relative read abundance of dominant gastrointestinal nematode families

**Model**	**Term**	**LR *χ*** ^**2**^	***df***	***P* value**
	Relative read abundance	11.59	1	<0.001
Trichostrongylidae model	Season	48.18	3	<0.001
	Population type	7.82	1	0.005
	Relative read abundance	11.60	1	<0.001
Molineidae model	Season	48.18	3	<0.001
	Population type	7.81	1	0.005

Each dominant nematode family was analysed in a separate additive model, with relative read abundance as the focal predictor and season and population type as covariates. Significance was assessed using LR tests. LR *χ*^2^, likelihood-ratio chi-square statistic; *df*, degrees of freedom.

In contrast, Molineidae exhibited substantially lower relative read abundance overall and showed a different seasonal pattern (Table 4). Its relative read abundance was highest in spring and lowest in summer, with intermediate values observed in autumn and winter. Comparisons between populations revealed significantly higher relative read abundance of Molineidae in wild gazelles during spring (*P* < 0.001), with no significant differences in summer (*P* = 1.000), autumn (*P* = 0.241) and winter (*P* = 1.000).

### Associations between faecal cortisol concentrations and dominant gastrointestinal nematode taxa

After accounting for season and population type, faecal cortisol concentrations were associated with Trichostrongylidae relative read abundance (LR *χ*^2^ = 11.59, *df* = 1, *P* < 0.001) and Molineidae relative read abundance (LR *χ*^2^ = 11.60, *df* = 1, *P* < 0.001). Season (*P* < 0.001) and population type (*P* = 0.005) were also significant predictors in both models (Table 5). Neither the relative read abundance × population type interaction for Trichostrongylidae nor the corresponding interaction for Molineidae was statistically supported (Trichostrongylidae: LR *χ*^2^ = 0.039, *df* = 1, *P* = 0.843; Molineidae: LR *χ*^2^ = 0.053, *df* = 1, *P* = 0.818; Table 6). Inclusion of the interaction terms did not improve model fit and resulted in slightly higher AIC values than the corresponding additive models (Trichostrongylidae: ΔAIC = 1.9; Molineidae: ΔAIC = 2.0). The more parsimonious additive models were therefore retained for interpretation.

**Table 6:** Tests of relative read abundance × population type interactions in Gamma GLMs of faecal cortisol concentrations

**Nematode family**	**Interaction term**	**LR *χ*** ^**2**^	***df***	***P* value**	**ΔAIC**
Trichostrongylidae	Relative read abundance × population type	0.039	1	0.843	1.9
Molineidae	Relative read abundance × population type	0.053	1	0.818	2.0

Relative read abundance of each dominant nematode family was modelled separately, with season and population type included as covariates. Interaction terms were assessed using LR tests, and model support was compared using AIC. LR χ^2^, likelihood-ratio chi-square statistic; *df*, degrees of freedom; ΔAIC, AIC of the interaction model minus that of the corresponding additive model.

The relative read abundances of Trichostrongylidae and Molineidae were nearly perfectly complementary across samples. This pattern was also evident in the two-panel scatterplots, with Trichostrongylidae generally occurring at high relative read abundance and Molineidae at correspondingly low relative abundance (Fig. 4a, b).

**Figure 4:**
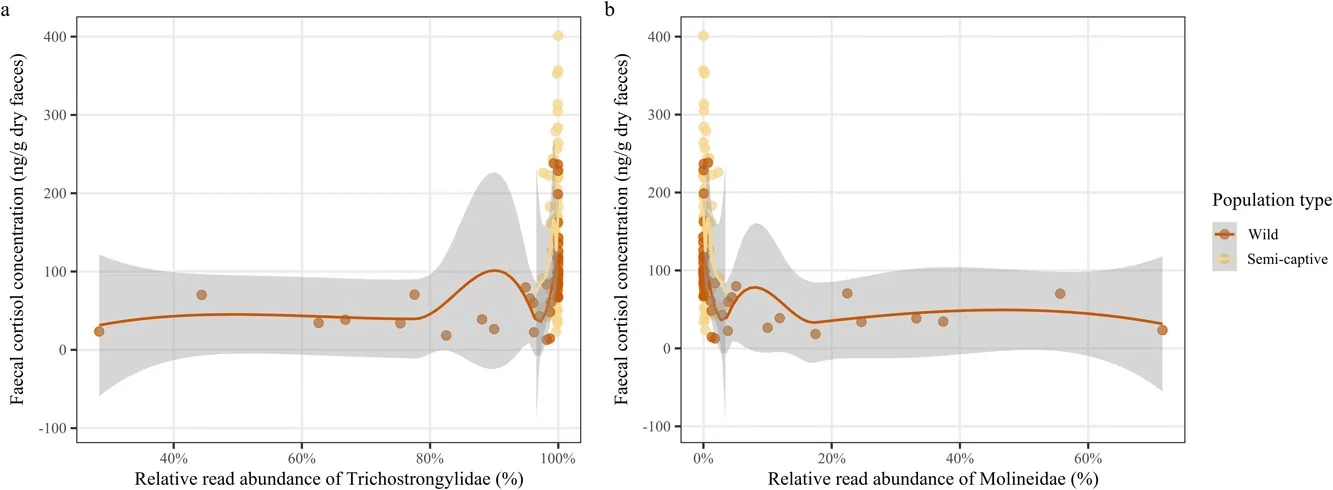
Exploratory visualization of faecal cortisol concentrations plotted against the relative read abundance of dominant gastrointestinal nematode families. (a) Trichostrongylidae relative read abundance (%) and (b) Molineidae relative read abundance (%). Points represent individual faecal samples from wild and semi-captive populations. Solid lines show LOESS curves for visualization purposes only, and shaded areas indicate 95% confidence bands (*n* = 115).

## Discussion

This study systematically investigated seasonal and population-level variations in faecal cortisol concentrations and gastrointestinal nematode communities in wild and semi-captive Przewalski’s gazelles, and further explored the association between host stress physiology and nematode dynamics. Seasonal variation was evident in both faecal cortisol concentrations and nematode community composition, with the most pronounced differences between populations occurring in spring. During this period, semi-captive individuals exhibited significantly higher cortisol levels and greater relative read abundance of Trichostrongylidae compared with their wild counterparts. Moreover, faecal cortisol concentrations were significantly associated with variation in dominant nematode community composition. Glucocorticoid concentrations in wild animals are shaped by multiple interacting intrinsic and extrinsic factors, including seasonal and environmental conditions, food availability, reproductive status, biotic interactions and anthropogenic disturbances (Pereira  *et al.*, 2006; Santicchia  *et al.*, 2018, 2020; Tranquillo  *et al.*, 2022). In Przewalski’s gazelles, faecal cortisol concentrations exhibited pronounced seasonal fluctuations, consistent with patterns reported in other ruminants, including red deer (*Cervus elaphus*) (Huber  *et al.*, 2003), cattle (*Bos taurus*) (Ogino  *et al.*, 2014), sheep (*Ovis aries*) (Snoj  *et al.*, 2014), Bedouin goats (*Capra aegagrus hircus*) (Chergui  *et al.*, 2017) and springbok (*Antidorcas marsupialis*) (Turner  and  Getz, 2010). However, temporal patterns differed between the two populations, as semi-captive gazelles exhibited their highest cortisol concentrations in spring, whereas wild gazelles exhibited the highest values in summer. These contrasting patterns suggest that stress physiology in Przewalski’s gazelles is closely linked to seasonal environmental variation but may be modified by differences in habitat conditions and management regimes. Elevated temperature and longer photoperiods have been reported to influence cortisol secretion through endocrine regulation in ungulates (Chergui  *et al.*, 2017). In contrast, lower temperatures and seasonal reductions in forage quality may alter energetic requirements and activity patterns in Przewalski’s gazelles, potentially contributing to variation in glucocorticoid concentrations across seasons. Such endocrine adjustments may represent adaptive physiological mechanisms that enable Przewalski’s gazelles to maintain physiological homeostasis under the pronounced environmental seasonality of the Qinghai–Tibet Plateau.

Differences in cortisol levels between wild and semi-captive populations were strongly season-dependent. During spring, semi-captive Przewalski’s gazelles displayed significantly higher faecal cortisol levels, a pattern widely documented in captive or managed mammals, including spotted hyenas (*Crocuta crocuta*) (Goymann  *et al.*, 2001; Benhaiem  *et al.*, 2012), cheetahs (*Acinonyx jubatus*) (Terio  *et al.*, 2004), fallow deer (*Dama dama*) (Konjević  *et al.*, 2011, 2016) and Canada lynx (*Lynx canadensis*) (Fanson  *et al.*, 2012). In the present study, higher cortisol levels and greater relative read abundance of Trichostrongylidae were observed in semi-captive individuals compared with wild gazelles during spring. This pattern may reflect the simultaneous influence of multiple factors, including dominant nematode community dynamics and environmental or management-related stressors, during this period. In summer, no significant population-level difference in cortisol was observed, likely reflecting favourable climatic conditions, abundant forage and reduced anthropogenic disturbance. In contrast, during autumn and winter, higher cortisol concentrations observed in wild gazelles may be associated with declining forage quality, intensified competition with livestock, and increased habitat pressure (Liu  and  Jiang, 2002). Under food limitation, ungulates typically shift to lower quality but more abundant forage (Emlen, 1968). Winter diets of Przewalski’s gazelles may include nutritionally poor plant species such as *Stellera chamaejasme*, *Iris* spp. and *Artemisia* spp. (Liu  and  Jiang, 2004). Such dietary shifts likely reduce energy acquisition efficiency and compromise physiological condition. In contrast, lower cortisol concentrations in semi-captive individuals during winter suggest that supplementary feeding and stable access to water may partially buffer seasonal nutritional stress.

In addition to environmental and management-related factors, dynamics of the gastrointestinal nematode community may also be associated with changes in glucocorticoid levels. The gastrointestinal nematode community of Przewalski’s gazelles was dominated by Trichostrongylidae and Molineidae, consistent with previous findings in wild and domestic ruminants, including Mongolian gazelles (*Procapra gutturosa*) and Tibetan sheep (*Ovis aries*) (Wang  *et al.*, 2016). The occurrence of similar nematode taxa among sympatric ungulates may reflect shared pasture use, faecal–oral transmission routes and opportunities for cross-species exposure (VanderWaal  *et al.*, 2014). Nematode eggs are shed in faeces and develop into infective larvae in the pasture environment, completing their life cycle when hosts ingest contaminated forage or water (Fiel  *et al.*, 2012). Spatial and dietary overlap with livestock may therefore increase opportunities for parasite transmission.

Marked seasonal variation was detected in nematode community composition. Trichostrongylidae reached its highest relative read abundance in summer, whereas Molineidae showed its highest relative read abundance in spring, indicating distinct seasonal representation among dominant nematode taxa. These seasonal differences may reflect variation among nematode taxa in environmental sensitivity and life history strategies. The free-living stages of trichostrongylid nematodes are highly sensitive to environmental conditions (O’Connor  *et al.*, 2006), with eggs and larvae generally developing more successfully under warm and moist environmental conditions (Anderson, 2000). Developmental arrest (hypobiosis) can allow gastrointestinal nematodes to persist through unfavourable periods and resume development when environmental conditions improve (Ash  and  Atkinson, 1983; Armour  and  Duncan, 1987; Jagdale  and  Grewal, 2003; Vlaar  *et al.*, 2021). Within the Przewalski’s gazelle nematode community, *Marshallagia dentispicularis* and *Ostertagia* sp. NMG-2010 were among the predominant taxa. Cold tolerance has been reported in closely related *Marshallagia* species, including *M. marshalli* (Carlsson  *et al.*, 2012, 2013; Kutz  *et al.*, 2012; Aleuy  *et al.*, 2019), whereas developmental arrest is well documented in *Ostertagia* spp. (Armour  and  Duncan, 1987). However, these mechanisms have not yet been directly evaluated in gastrointestinal nematodes infecting Przewalski’s gazelles. Seasonal precipitation (O’Connor  *et al.*, 2008) and temperature (Paull  *et al.*, 2012; Mordecai  *et al.*, 2013) may further regulate transmission by affecting the survival and development of free-living stages. Similar climate-associated patterns have been reported in other ruminants, including red deer (*Cervus elaphus*) (Albery  *et al.*, 2018). The alpine continental climate of the Qinghai–Tibet Plateau, with long, cold winters and short, cool, relatively humid summers, may therefore contribute to the pronounced seasonal structure observed in the nematode community. Overall, the seasonal dynamics of gastrointestinal nematode communities in Przewalski’s gazelles appear to reflect the combined influence of climatic seasonality, host ecology and environmental conditions in this high-altitude ecosystem.

Importantly, the relative read abundances of Trichostrongylidae and Molineidae were nearly perfectly complementary across samples. Their contrasting associations with cortisol should therefore not be interpreted as independent family-specific effects, but rather as opposite expressions of the same nematode-community compositional gradient. An increase in the relative representation of one dominant family was accompanied by a corresponding decrease in the other. Accordingly, the observed relationship with cortisol is more appropriately interpreted as an association between host stress physiology and shifts in nematode community composition. Moreover, the relative read abundance × population type interactions were not statistically supported, providing no statistical evidence that this association differed between wild and semi-captive populations.

The seasonal coincidence between host stress physiology and nematode community composition was most apparent in spring, when semi-captive gazelles exhibited higher cortisol concentrations together with a shift in nematode composition towards greater Trichostrongylidae representation relative to wild gazelles. However, given the absence of a significant interaction with population type, these concurrent differences should not be interpreted as evidence of a stronger parasite–cortisol relationship in semi-captive animals. Rather, spring may represent a period in which multiple physiological and environmental pressures converge, with increased energetic demands following winter coinciding with limited forage availability during early regrowth (Parker  *et al.*, 2009; Anderwald  *et al.*, 2021), alongside potential parasite-associated physiological costs. Parasites may increase metabolic costs by diverting nutrients and stimulating immune responses (Sykes  and  Greer, 2003; Shanebeck  *et al.*, 2022; Douhard  *et al.*, 2025), potentially increasing trade-offs between somatic recovery and immune defence (Coop  and  Kyriazakis, 1999). Environmental conditions in semi-captive systems may additionally influence parasite exposure through factors such as local host density, faecal accumulation and microenvironmental conditions (Ezenwa, 2004a, 2004b). Such factors could modify transmission opportunities, but their effects were not quantified directly in this study.

Previous studies in wild birds, experimental mammals and meta-analyses across vertebrates have likewise reported associations between parasitism and glucocorticoid responses (Raouf  *et al.*, 2006; O’Dwyer  *et al.*, 2020; Barros-Gonçalves  *et al.*, 2021). Elevated cortisol secretion may contribute to the mobilization of energy reserves required to maintain homeostasis under challenging physiological and environmental conditions (Wingfield  and  Romero, 2000; Kitaysky  *et al.*, 2001). Sustained glucocorticoid elevation can also modulate immune function (Schoenle  *et al.*, 2019; Shimba  *et al.*, 2021), including aspects of cell-mediated immunity (Elenkov, 2004; Franchimont, 2004), suggesting a potential physiological pathway linking endocrine and parasite processes. Nevertheless, these mechanisms were not directly tested here. The present findings therefore support a seasonal association between host stress physiology and nematode community composition, while the direction and mechanisms of these relationships remain to be established.

In conclusion, our findings reveal pronounced seasonal associations between gastrointestinal nematode dynamics and stress physiology in *P. przewalskii*. Spring emerged as a period in which differences in cortisol concentrations and nematode community composition between wild and semi-captive gazelles were particularly apparent, potentially reflecting the convergence of multiple physiological and environmental pressures. By integrating endocrine and parasitological measures across contrasting population contexts, this study advances understanding of seasonal stress–parasite associations in an alpine ungulate. Continued longitudinal studies integrating physiological and parasitological metrics will improve understanding of how environmental variability relates to stress–parasite interactions and may provide valuable insights for the conservation and management of threatened wildlife populations.

## Supplementary Material

Web_Material_coag081

## Data Availability

The data underlying this article are available from the corresponding author upon reasonable request.
