# Supplementary material for: Seasonal coupling between stress physiology and gastrointestinal nematode communities in wild and semi-captive populations of an endangered ungulate
Source: Conserv Physiol. 2026 Oct 22;14(1):coag081. doi: 10.1093/conphys/coag081 (PMC13688043; doi:10.1093/conphys/coag081)
Supplement: Supplementary file 1 [file web_material_coag081.zip › Supplementary Material.pdf]

Supplementary Material

Seasonal coupling between stress physiology and gastrointestinal nematode communities in wild and semi-captive populations of an endangered ungulate

Ming LU, Ya-Lun WANG, Han-Qing LIU, Xiao-Fan MA, Yong-Shen WANG, Yong-Gang NIE*

Supplementary Tables

Supplementary Table S1. Within-season pairwise comparisons of faecal cortisol concentrations between wild and semi-captive populations.

Season	Contrast	Ratio	SE	*df*	*t* ratio	*P* value
Spring	Wild / Semi-captive	0.216	0.028	199	-11.984	< 0.001
Summer	Wild / Semi-captive	1.044	0.111	199	0.407	0.684
Autumn	Wild / Semi-captive	1.460	0.168	199	3.281	0.001
Winter	Wild / Semi-captive	1.295	0.163	199	2.053	0.041

Note: Pairwise comparisons were performed using estimated marginal means derived from the Gamma generalized linear model. Ratios are expressed as Wild / Semi-captive; values < 1 indicate lower estimated faecal cortisol concentrations in wild populations, whereas values > 1 indicate higher estimated concentrations in wild populations. SE, standard error; *df*, degrees of freedom.

Supplementary Table S2. Effects of environmental covariates and biological factors on faecal cortisol concentrations based on an environmentally adjusted Gamma generalized linear model.

Term	LR χ^2	*df*	*P* value
Population type	0.54	1	0.462
Season	20.27	3	< 0.001
Sex	1.48	1	0.224
Standardized NDVI	0.96	1	0.328
Standardized temperature	0.60	1	0.439
Standardized precipitation	2.81	1	0.094
Population type × season	117.04	3	< 0.001

Note: The model used a Gamma error distribution with a log link function. NDVI (normalized difference vegetation index), temperature, and precipitation were standardized prior to analysis. Significance was assessed using likelihood-ratio tests. LR χ^2, likelihood-ratio chi-square statistic; *df*, degrees of freedom.
